# Author Correction: Restoration cannot be scaled up globally to save reefs from loss and degradation

**DOI:** 10.1038/s41559-025-02758-9

**Published:** 2025-05-29

**Authors:** Clelia Mulà, Corey J. A. Bradshaw, Mar Cabeza, Federica Manca, Simone Montano, Giovanni Strona

**Affiliations:** 1 Faculty of Biological and Environmental Sciences, Organismal and Evolutionary Biology Research Programme, University of Helsinki, Helsinki, Finland; 2 Australian Research Council Centre of Excellence for Australian Biodiversity and Heritage, Wollongong, New South Wales Australia; 3 Australian Research Council Centre of Excellence for Indigenous and Environmental Histories and Futures, Cairns, Queensland Australia; 4 Global Ecology, Partuyarta Ngadluku Wardli Kuu, College of Science and Engineering, Flinders University, Adelaide, South Australia Australia; 5 Department of Earth and Environmental Sciences, University of Milano-Bicocca, Milan, Italy; 6 Marine Research and High Education Center, Magoodhoo Island, Republic of Maldives; 7 European Commission, Joint Research Centre, Ispra, Italy

**Keywords:** Conservation biology, Restoration ecology

Correction to: *Nature Ecology & Evolution* 10.1038/s41559-025-02667-x, published online 8 April 2025.

This article was originally published under standard Springer Nature license (© The Author(s), under exclusive licence to Springer Nature Limited). It is now available as an open-access article under a Creative Commons Attribution 4.0 International license, © The Author(s). The error has been corrected in the HTML and PDF versions of the article.

