# Supplementary material for: Restoration cannot be scaled up globally to save reefs from loss and degradation
Source: Nat Ecol Evol. 2025 Apr 8;9(5):822–32. doi: 10.1038/s41559-025-02667-x (PMC12122368; doi:10.1038/s41559-025-02667-x)
Supplement: Supplementary file 1 — Supplementary Figs. 1 and 2. [file 41559_2025_2667_MOESM1_ESM.pdf]

nature ecology & evolution

Supplementary information https://doi.org/10.1038/s41559-025-02667-x

Restoration cannot be scaled up globally to save reefs from loss and degradation

In the format provided by the authors and unedited

Supplementary Information

Restoration cannot be scaled up globally to save reefs from loss and degradation

Clelia Mula[1], Corey J. A. Bradshaw[2,3,4], Mar Cabeza[1], Federica Manca[1], Simone Montano[5,6], Giovanni Strona[7]*

*giovanni.strona@ec.europa.eu

[1]Faculty of Biological and Environmental Sciences, Organismal and Evolutionary Biology Research Programme, University of Helsinki, PO Box 65 Viikinkaari 1, Helsinki, 00014 Finland [2]Global Ecology | *Partuyarta Ngadluku Wardli Kuu*, College of Science and Engineering, Flinders University, GPO Box 2100, Adelaide, South Australia 5001, Australia

[2]Australian Research Council Centre of Excellence for Australian Biodiversity and Heritage, Wollongong, New South Wales, Australia

[3]Australian Research Council Centre of Excellence for Indigenous and Environmental Histories and Futures, Cairns, Queensland, Australia

[4]Global Ecology | Partuyarta Ngadluku Wardli Kuu, College of Science and Engineering, Flinders University, GPO Box 2100, Adelaide, South Australia 5001, Australia

[5]Department of Earth and Environmental Sciences (DISAT), University of Milano-Bicocca, Piazza Della Scienza 1, 20126, Milan, Italy

[6]MaRHE Centre (Marine Research and High Education Center), Magoodhoo Island, Faafu Atoll, 12030, Republic of Maldives

[7]European Commission, Joint Research Centre (JRC), Ispra, Italy

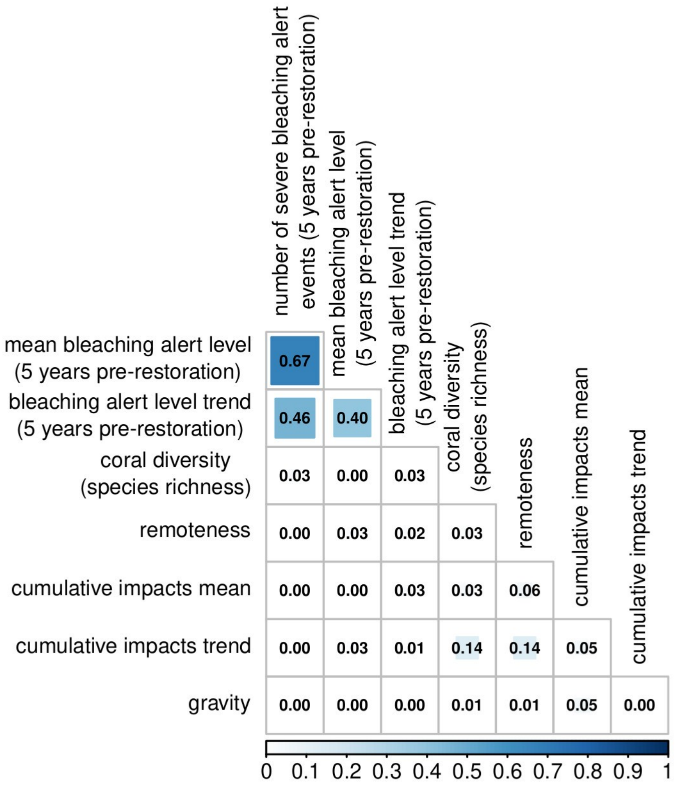

Figure S1 | Pairwise correlation (Pearson's R^2) between the independent variables used to predict the choice of restoration sites. Total n observations = 3324.

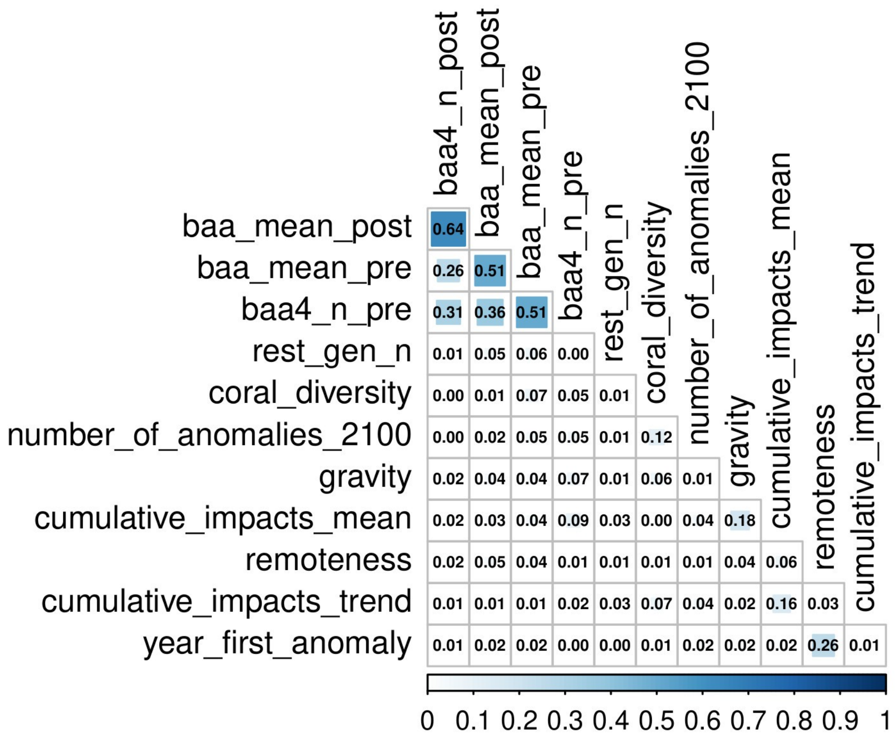

Figure S2 | Pairwise correlation (Pearson's R2) between the independent variables used to predict coral restoration success. Total n observations = 134.
